# Climatic-driven seasonality of emerging dengue fever in Hanoi, Vietnam

**DOI:** 10.1186/1471-2458-14-1078

**Published:** 2014-10-16

**Authors:** Thi Thanh Toan Do, Pim Martens, Ngoc Hoat Luu, Pamela Wright, Marc Choisy

**Affiliations:** Biostatistics and Medical Informatics Department, Institute of Training for Preventive Medicine and Public Health, Hanoi Medical University, Hanoi, Vietnam; International Centre for Integrated assessment and Sustainable development, Maastricht University, Maastricht, The Netherlands; The Medical Committee Netherlands-Vietnam, Amsterdam, The Netherlands; MIVEGEC (IRD 224-CNRS 5290-Université Montpellier 1 et 2), Centre IRD, Montpellier, France; Oxford University Clinical Research Unit, Hanoi, Vietnam

**Keywords:** Dengue fever, Seasonality, Emergence, Climatic factors, Hanoi, Vietnam

## Abstract

**Background:**

Dengue fever (DF) has been emerging in Hanoi over the last decade. Both DF epidemiology and climate in Hanoi are strongly seasonal. This study aims at characterizing the seasonality of DF in Hanoi and its links to climatic variables as DF incidence increases from year to year.

**Methods:**

Clinical suspected cases of DF from the 14 central districts of Hanoi were obtained from the Ministry of Health over a 8-year period (2002–2009). Wavelet decompositions were used to characterize the main periodic cycles of DF and climatic variables as well as the mean phase angles of these cycles. Cross-wavelet spectra between DF and each climatic variables were also computed. DF reproductive ratio was calculated from Soper’s formula and smoothed to highlight both its long-term trend and seasonality.

**Results:**

Temperature, rainfall, and vapor pressure show strong seasonality. DF and relative humidity show both strong seasonality and a sub-annual periodicity. DF reproductive ratio is increasing through time and displays two clear peaks per year, reflecting the sub-annual periodicity of DF incidence. Temperature, rainfall and vapor pressure lead DF incidence by a lag of 8–10 weeks, constant through time. Relative humidity leads DF by a constant lag of 18 weeks for the annual cycle and a lag decreasing from 14 to 5 weeks for the sub-annual cycle.

**Conclusion:**

Results are interpreted in terms of mosquito population dynamics and immunological interactions between the different dengue serotypes in the human compartment. Given its important population size, its strong seasonality and its dengue emergence, Hanoi offers an ideal natural experiment to test hypotheses on dengue serotypes interactions, knowledge of prime importance for vaccine development.

## Background

Dengue Fever (DF) has recently been recognized by WHO as the fastest spreading tropical disease across all continents. Bhatt et al. [1] estimated that the global number of new infections per year (390 millions, 95% confidence interval: 284–528) is largely underestimated: only 96 millions (95% confidence interval: 67–136) being declared yearly. DF is one of the many symptoms (ranging from mild fever to hemorrhagic fever and shock syndrome) caused by one of the four serotypes of dengue virus (Flaviridae family). Even though recovery from dengue confers life-long immunity against the infecting serotype, immunological interactions between the different serotypes are not fully understood. The virus is transmitted by bites of female *Aedes aegypti* or *albopictus* mosquitoes in the inter-tropical regions of the world. In absence of vaccine (under development), mosquito control is the only available method of prophylaxy.

In Vietnam, dengue is recognized as a major cause of mortality and morbidity and ranks amongst the top ten communicable diseases in terms of overall health burden [2]. All four dengue virus serotypes have been found circulating in Vietnam with the dominant one varying over time. Reports from the National Institute of Hygiene and Epidemiology show that DENV-1 and DENV-2 have been the predominant circulating viruses almost every year. DENV-3 emerged in the late 1990s and was responsible for the large outbreak of 1998, whereas DENV-4 was also detected between 1999 and 2003 [3]. Dengue transmission occurs throughout the year in Vietnam, with peaks in the numbers of cases (72% of total cases) reported between June and November [4]. There are regional variations in the seasonality of dengue epidemiology in Vietnam. In the Northern and Central Highland regions, dengue notifications are low during the winter time from December to March, while in southern regions, dengue transmission occurs throughout the year, even if it sharply increases during the rainy season from July to September. Given that dengue is a vector-born disease and that vector population dynamics are strongly dependent on climatic factors, the diversity of climates in Vietnam may explain the observed diversity of dengue epidemiological dynamics.

Hanoi, the capital of Vietnam, is located in the North of the country and known as a low transmission setting of DF [5]. Hanoi experiences annual seasonal dengue outbreaks with the pinnacle of epidemics usually falling in September/October and ending in November/December. Over the last decade, the number of DF cases has been increasing from year to year, reaching a peak in 2009. According to the Ministry of Health’s statistics, the outbreak in Hanoi in 2009 is the most important outbreak of the last decade, with 384 notified cases per 100,000 individuals. Interestingly, 2009 was also the year El Niño increased actively [6, 7]. There are only few studies published on dengue epidemiology in Hanoi that are based on the public health surveillance data routinely collected through the Ministry of Health’s notifiable diseases surveillance program. Toan et al. [8] show that there are spatio-temporal clusters of DF limited to a radius of 1,000 m and a duration of 29 days. This study also demonstrates that most of the DF cases occur between June and November, during which the rainfall and temperatures are highest. Cuong et al. [5] use wavelet analysis to relate dengue incidence to climatic variables and suggest that all the tested local climatic variables (total rainfall; mean wind velocity; mean, maximum and minimum temperatures; relative humidity) are significantly associated with dengue incidence around the annual periodicity: on average, dengue incidence follows the seasonal peak of rainfall and mean temperature with a lag of 1 to 2 months.

Other studies have been carried out on the correlation between climate and DF in other parts of Vietnam as well as in other parts of the world, using a wide spectrum of mathematical and statistical modeling methods [6, 9–25]. In Vietnam, most of the studies have been carried out in the south and the center of the country, and showed significant associations between climatic variables and dengue incidence. A wavelet analysis of monthly dengue cases from the province of Binh Thuan has shown a non-stationary relationship between El Niño Southern Oscillation indice and dengue incidence in the 2–3 year periodic band [6]. Meanwhile, a correlation study carried out on monthly dengue cases from the province of Daklak has found the risk of dengue to be associated with high temperature, high relative humidity and rainfall, but inversely associated with duration of sunshine [22]. Findings from other studies in many other parts of the world also show climatic variables to have an effect on dengue transmission. Studies in Thailand [11], Barbados [12], Taiwan [16], Guangzhou, China [18], the French West Indies [21] and in Colombia [26] showed a positive correlation between dengue incidence and minimum and maximum temperatures, precipitation and relative and absolute humidities. However, depending on the approach of analysis and the areas, these correlations were more or less strong. In Barbados, the strongest correlation was found at a lag of 6, 12 and 16 weeks for vapour pressure and minimum and maximum temperatures respectively, whereas in Taiwan the highest correlations were found with maximum temperature at a lag of 5 weeks and with total precipitation at a lag of 7 weeks. In Colombia, Eastin et al. [26]‘s results suggest that DF cases increase 2 to 5 weeks after the daily temperature range remains for an extended period within the temperature range optimal for vector survival and disease transmission. Nagao et al. [11] in Thailand and Yi et al. [27] in Guangdong, China, demonstrated that the distributions of *Aedes* species and dengue cases were positively associated with high absolute humidity, which itself increases with high temperature and rainfall. In San Juan, Puerto Rico, Schreiber [9] used a water budgeting technique and showed that high levels of dengue are associated with reduced actual evapotranspiration, minimum temperature and with high levels of precipitation. In Taiwan, using autoregressive integrated moving average models, Wu et al. [20], found a negative association of dengue incidence with temperature and relative humidity. Finally, in the city of Noumea (New Caledonia), Descloux et al. [24] recently documented a high seasonality of dengue incidence, with an epidemic peak (March-April) lagging the warmest temperature by 1 to 2 months and in phase with maximum precipitations, relative humidity and entomological indices.

In the present study, we consider vapor pressure and relative humidities, temperature and rainfall in order to identify which of these variables are most critical for the onset of dengue epidemics. Compared to Cuong et al. [5], who also investigated the links between dengue and climatic variables in Hanoi, Vietnam, we here consider vapor pressure in addition to relative humidity. Vapor pressure is a measure of absolute humidity and this climatic variable is often neglected in the studies investigating the links between climate and disease transmission, even though it has been proved to play a role more important than relative humidity for the transmission of some diseases such as influenza (e.g. [28]). A second difference with Cuong et al. [5] is that we here work on weekly incidence aggregates instead of monthly aggregates. With this finer temporal resolution we aim at investigating intra-annual patterns of seasonality. We first quantify the synchrony between weekly dengue incidence and the four climatic variables between 2002 and 2009. We then estimate the values of reproductive ratio of dengue fever through time and characterize the trend and seasonality of reproductive ratio. We finally discuss the results in light of immunological and entomological factors specific to dengue epidemiology in Hanoi, Vietnam.

## Methods

Setting: Hanoi (1,760 km^2^) is located in the Red River Delta, in the centre of North Vietnam (21^o^ 2 ’ N, 105° 51 ’ E). The city experiences the typical climate of northern Vietnam, where summers are hot and humid, and winters are, by national standards, relatively cold and dry.

### Data collection

The data used in this study have been previously published by Cuong et al. [5] and Toan et al. [8]. Clinical suspected cases of DF in old Hanoi (14 districts of Hanoi before merging with Ha Tay in 2008) are reported to the surveillance system of Hanoi Center for Preventive Health. The criteria for notification of DF disease are based on the guidelines of the Ministry of Health (2006) on surveillance, diagnosis and treatment of dengue, in which suspected dengue cases are based on acute febrile illness (≥38°C) of 2–7 days duration with at least two of the following non-specific manifestations of dengue fever: headache, retro-orbital pain, myalgia, arthralgia, rash, hemorrhagic manifestations, and leucopenia [29]. The data analyzed here include all reported cases from January 2002 to December 2009, aggregated by week.

Daily weather data for Hanoi from 2002 to 2009 were provided by the National Centre for Hydrometeorological Forecasting. They include the records of mean, maximum, and minimum temperatures (T, in°C), rainfall (in mm) and relative humidity (in %).

### Vapor pressure

We used vapor pressure (VP, in mb) as a proxy of absolute humidity. VP was calculated from relative humidity (RH, in %) and temperature T using the Clausius–Clapeyron formula [28, 30]:
1

where L = 2,257 J/g is the latent heat of evaporation for water, *R*_*v*_ = 416.5 J/(kg K) is the gas constant for water vapor, *T*_*0*_ *= 273.15 K* and *VP*_*0*_ *= 6.11 mb* is the vapor pressure at which water would change phase between vapor and liquid if the temperature was *T*_*0*_. Note that in the above formula temperature T is expressed in °C instead of K as in Shaman and Kohn [28].

Climatic variables were aggregated by week using sums for rainfall and mean values for all the other variables.

### Wavelet analysis

**Figure 1:**
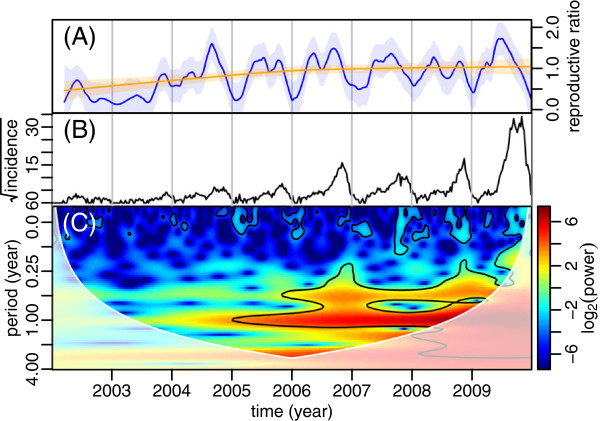
**The reproductive ratio, time series and wavelet power spectrum of DF in Hanoi (14 districts) from 2002 to 2009. (A)** The reproductive ratio was estimated from equation 2 (see text) and smoothed by lowest regressions with smoothing factors equal to 0.05 (blue) and 0.90 (red). The shaded areas around the lines represent the 95% confidence intervals calculated assuming a normal distribution of errors. **(B)** Time series of the square-root transformed weekly DF incidence. **(C)** Wavelet power spectrum of the square-root transformed weekly DF incidence. The black contour lines show the regions of power significant at the alpha-risk of 0.05. The paled region of the spectrum delineates the cone of influence due to the zero-padding of the time series. The power increases from dark blue to dark red.

Epidemiological data can be substantially non-stationary [31–33] as is the case for dengue in Hanoi where it is emerging (see in particular the increase in mean and amplitude over time on Figure 1B). Here we performed wavelet decomposition, a time-series statistical analysis allowing to efficiently deal with non-stationary data. Specifically, we used the Morlet wavelet [34], classically used in ecology with a non-dimensional frequency ω_0_ = 6. An advantage of using the Morlet wavelet is that it is a complex wavelet, allowing to quantify the phase and thus calculate time lags between different time series.

Coherence based on wavelets allows to perform similar analysis as cross-correlation but for potentially non-stationary signals. Wavelet coherences were calculated to examine the association between two time series, both in time and frequency. Coherence spectra allow to investigate whether different periodic modes of two time series tend to oscillate simultaneously and, if yes, to identify the periodicity around which this association takes place.

Significance levels were calculated by a Chi-square test assuming that the wavelet coefficients are normally distributed as described in Torrence and Compo [35]. The detailed theory for wavelet analysis has been described elsewhere [4]. Before wavelet decomposition, time series were square-root transformed in order to mitigate the weights of high values. They were also zero-padded to the next power of 2 of their length (i.e. 512), in order to minimize edge effects [35].

### Reproductive ratio

The reproductive ratio R is the expected number of infections caused by one infected individual. It is maximal at the start of an epidemic when the host population is fully susceptible and then decreases over the course of the epidemics. It reaches the equilibrium value of 1 at epidemic peak and decreases below 1 after it. The initial and maximum value of the reproductive ratio is called the basic reproductive ratio R_0_ and is the classical epidemiological statistics as its value relative to 1 informs about the potential for an epidemic to occur.

We followed Soper [36] as reported in Keeling and Rohani [37] and approximated the reproductive ratio R by:
2

where *C*_*t+1*_ and *C*_*t*_ are the numbers of cases at times *t + 1* and *t* respectively and *α* is a parameter that expresses the infection generation length (i.e. the sum of the infectious and latent periods) in the same units as the data time steps (here 1 week). The infection generation length for dengue being approximately 2 weeks [38], we set this parameter *α* to the value of 2. The other part of Soper [36]’s formula expresses the reproductive ratio as a function of the number of susceptibles in the population:
3

where X* and *X*_*t+1*_ are the numbers of susceptibles before the epidemics and at time *t + 1*, respectively and k is a parameter reflecting some potential external forcings (such as climatic ones). This latter part of the Soper [36]‘s equation shows that variations in the reproductive ratio are due either to variations in the number of susceptibles in the population, or to some external forcings directly affecting the transmissibility of the disease (in our case climatic factors acting on the vector population dynamics and density). Given that dengue is emerging in Hanoi and that the basic reproductive ratio of dengue is generally low [38], we expect the depletion of susceptible in the population to be very slow. We expect it to be even slower given the fact that dengue can actually be caused by four different serotypes with no permanent cross-immunity between them. Hence, among the two above-cited factors that can affect the seasonality of the reproductive ratio (susceptible depletion and external forcing), we assume that susceptible depletion is negligible before any external forcing such as climatic drivers on the mosquito population dynamics.

Time series of the calculated seasonal factor *k* were smoothed by lowess regression with smoothing factors equal to 0.05 and 0.90 in order to reveal its seasonality and its long-term trend respectively. Confidence intervals were calculated by assuming normal distribution of errors.

Given the uncertainty on the infection generation length and the assumption made on the number of susceptibles, estimates of the reproductive ratio will be treated with caution: only their trend and seasonality will be interpreted, not their absolute values, on which we will have limited confidence.

All analyses were conducted in R (R Core Team [39]) and wavelet analyses were performed using the “biwavelet” R package [40].

## Results

### Dengue incidence and its reproductive ratio in Hanoi

From 2002 to 2009, 23,195 DF cases were reported in Hanoi with the average annual incidence rate of 69.22/100,000. Overall, the incidence of DF increased over the 8 years of the study, with a sharp increase during the period 2005–2009. This period of increasing incidence is visible on the wavelet spectrum of Figure 1C. The highest peak of 3,697 cases was recorded in September 2009. Over the 5 years from 2005 to 2009, annual (1 year) and sub-annual (6 months) periodicities were significant. These two periodicities correspond to a slow increase of DF incidence from the beginning of the year to weeks 22–24 (June), followed by a rapid increase of incidence until weeks 44–46 (November) which ends by a sharp decrease in incidence at the end of the rainy season.

Figure 1A shows a long-term increase in the reproductive ratio (orange curve) as well as a non-stationary sub-annual periodicity with two peaks of this reproductive ratio per year (blue curve). These peaks are of roughly equal magnitude from 2005 to 2008, the second peak is substantially higher than the first one in 2004 and 2009, and the number of cases are too low before 2004 for any clear pattern to be visible (large confidence intervals on Figure 1B).

### Meteorological variables in Hanoi

**Figure 2:**
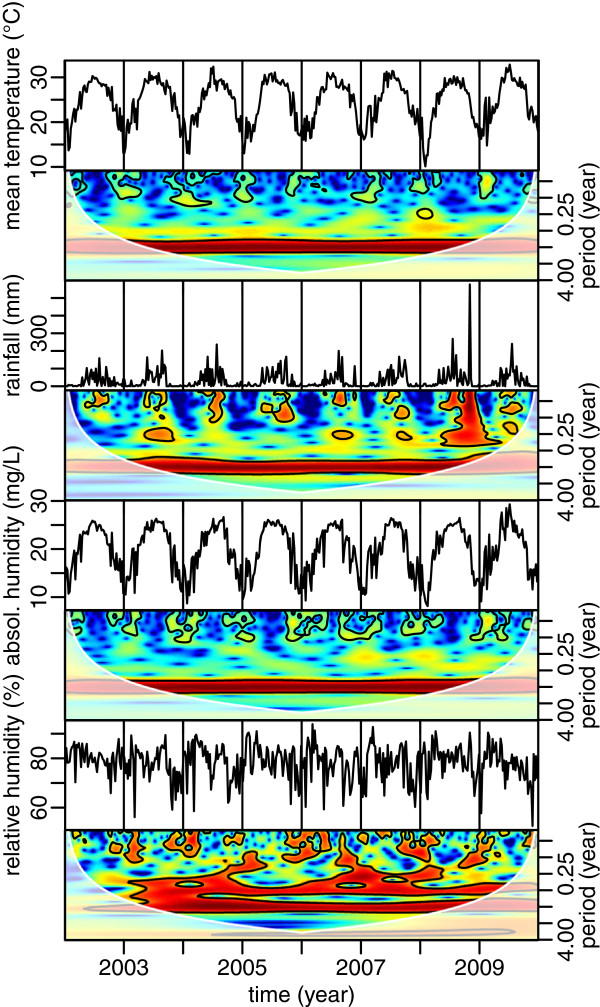
**Time series and wavelet power spectra of mean temperature, cumulative rainfall and mean absolute and relative humidities in Hanoi, from 2002 to 2009.** The black contour lines show the regions of power significant at the alpha-risk of 0.05. The paled region of the spectrum delineates the cone of influence due to the zero-padding of the time series. The power increases from dark blue to dark red.

The wavelet power spectra of temperature, precipitation, and humidities (relative humidity and vapor pressure) in Hanoi during the study period are shown in Figure 2. Temperature, precipitation and vapor pressure in Hanoi show significant annual periodicities that are constant through time, whereas relative humidity shows both annual and sub-annual periodicities, as observed on the DF incidence time series (Figure 1B).

**Figure 3:**
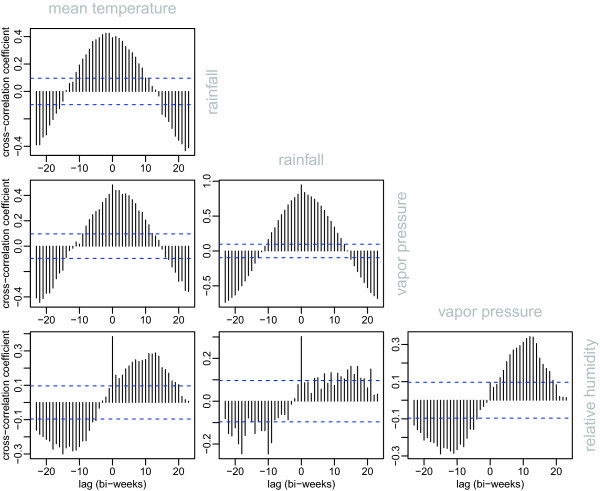
**Cross-correlation between the 4 climatic variables: mean temperature, rainfall, vapor pressure and relative humidity.** Horizontal blue dotted lines materialize the significativity thresholds at p = 0.05.

The cross-correlation coefficients among four climatic variables in Hanoi are presented in Figure 3. It shows that mean temperature, rainfall and vapor pressure are much correlated and in phase, whereas there is a significant lag between these three variables and relative humidity.

Over the 8 years of the study, the average temperature in Hanoi was found to be lowest in January and February (16.07 ± 1.65°C standard deviation) and highest in June and July (29.80 ± 0.47°C). Over the studied period, the coolest temperature was observed in week 5 (February) of 2008 and the warmest was observed in week 27 (July) of 2009 (10.11°C and 32.84°C respectively). July to September was the time of the year receiving the majority of the annual rainfall (271.9 ± 111.9 mm in average over one week). Week 44 (November) in 2008 recorded a rainfall extreme of 576.6 mm brought by the typhoon Maysak (Center for Excellence in Disaster Management and Humanitarian Assistance, [41]). The relative humidity in Hanoi is quite high (78.62 ± 4.28%), and is usually higher in February and March (cool but very rainy: 84.12 ± 2.56), and August and September (drier but very hot: 79.81 ± 3.54), than the rest of the year (76.80 ± 3.99).

### Coherences between meteorological variables and DF incidence in Hanoi

**Figure 4:**
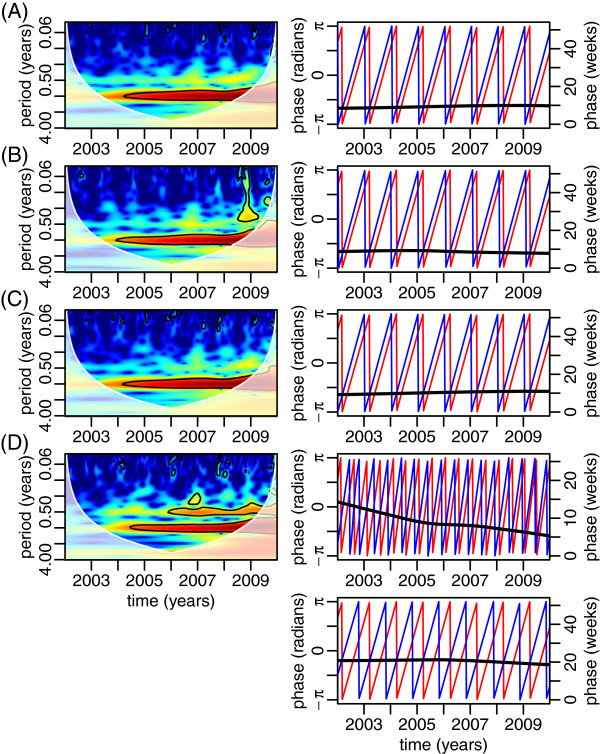
**Cross-wavelet power spectra between DF and mean temperature (A), rainfall (B) and absolute (C) and relative (D) humidities in Hanoi from 2002 to 2009 (left column).** The right column shows the phase angles of the climatic variables (blue, left y-axis) and DF (red, left y-axis), as well as their difference (black, right y-axis). These phase angles are calculated on signals that have been filtered around the period of maximal power in the spectra of the left column, i.e. annual periodicity for all the climatic variables, as well as also the semi-annual periodicity for the relative humidity. In spectra of the left column, the black contour lines show the regions of power significant at the alpha-risk of 0.05, the paled region of the spectrum delineates the cone of influence due to the zero-padding of the time series, and the power increases from dark blue to dark red.

Results of wavelet coherences between DF incidence and climate variables are shown in Figure 4. Significant coherences were observed between DF incidence and temperature, precipitation and humidity for the annual periodicity from approximately 2005 to 2009 (Figure 4A-D). Moreover a weaker, but still significant association between relative humidity and DF incidence was also seen for the sub-annual periodicity from 2006 to 2009 (Figure 4D).

In analyzing the phase difference at the annual cycle between DF incidence and the climatic variables, we found that dengue incidence was consistently trailing temperature, rainfall, vapor pressure and relative humidities with a delay of 9.37 ± 0.02 (standard error), 8.71 ± 0.02, 10.29 ± 0.03, and 18.05 ± 0.24 weeks respectively (see Figure 4). Interestingly, when looking at the statistical association between dengue incidence and relative humidity for the sub-annual cycle, it appears that the time delay of dengue incidence compared to relative humidity decreases from 14.30 to 5.27 weeks over the 8 years of the study.

## Discussion and conclusion

Using monthly aggregated data, Cuong et al. [5] showed a clear annual cycle for dengue transmission in Hanoi from 1998 to 2009. In the present study, using weekly data allowed us to further characterize a sub-annual periodicity, in addition to the annual one. This sub-annual periodicity is reflected in the DF incidence time series, by a slow increase of incidence from the beginning of the year to the weeks 22–24 (June), followed by a faster increase of incidence until weeks 44–46 (November), which ends by a sharp decrease of incidence at the end of the rainy season. A potential drawback of working on weekly instead of monthly data is that it decreases the incidence values and thus increases the noise. However, given that we can still detect clear periodicities in our wavelet spectra, this does not seem to affect our analysis too much.

When characterizing the reproductive ratio throughout the studied period, it displays, most of the years, two peaks per year, which is in accordance with the sub-annual periodicity of DF incidence. Among the climatic variables that we investigated (temperature, rainfall, relative humidities and vapor pressure), all of them expectedly displayed strong annual periodicities with temperature, rainfall and vapor pressure leading DF incidence by a constant delay of 8 to 10 weeks. In addition to this strong annual periodicity, relative humidity displays a sub-annual periodicity, as observed on DF incidence. The annual periodicity of relative humidity leads the annual periodicity of DF incidence by a constant delay of 18 weeks whereas the sub-annual periodicity of relative humidity leads the sub-annual periodicity of DF by a delay that decreases from 14.30 weeks in 2002 to 5.27 weeks in 2009 at an almost constant rate of 1.13 week per year. These results are in general agreement with the findings of other studies that climatic factors play a role in the transmission cycles of DF. Interestingly, these two incidence peaks per year that we observed in Hanoi with periods of low incidence occurring in January and February (the coldest months in Hanoi) and in June and July (the warmest months in Hanoi) are in accordance with Eastin et al. [26]‘s observation in Columbia where they noted a significant decreases of DF cases soon after extended periods of either very cool or very hot temperatures. Likewise, a study in Taiwan found three turning points of DF that occurred around early August, late August/early September, and late October/early November. The first two turning points were shown to relate with two typhoons around early to mid August in Taiwan that witnessed a sharp drop in temperature and substantial rainfall after it [16]. Similarly, other studies in Thailand and Singapore also revealed significant associations between climatic variables and dengue incidence ([13, 14, 42]; Tipayamongkholgul [43, 44]). For example, Tipayamongkholgul [43] conducted a study in the Gulf of Thailand and showed that the monthly average local relative humidity in the previous 3–6 months was negatively associated with epidemics of dengue and incidence of dengue cases. Woongkon et al. [44] in Chiang Rai, Thailand, showed that all climatic factors including minimum, maximum temperature, minimum and average relative humidity, evaporation, wind speed and rainfall lead increasing DF incidence by 0–2 months.

In our study, DF incidence is characterized by 2 quasi-cycles with a periodicity of 6 months with the first one showing a slow and constant increase and the second one showing a marked epidemic. The sharp rupture between these phases can be explained by the fact that the reproductive ratio is not constant throughout the year but actually exhibits two peaks per year, with the second peak at least as high as the first one. Given that dengue is vector-born, the factor limiting its transmission is either due to the mosquito population (mostly its population size), or the human population (mostly its proportion of susceptibles). Winter climatic conditions in Hanoi are not favorable to adult mosquitoes and most of the mosquito population survive the winter either as larvae or eggs [45]. In the spring, when weather conditions become favorable again, eggs hatch and adults emerge, probably causing the first peak on the reproductive ratio and the consequent DF incidence increase. The second peak on the reproductive ratio could be due to the second mosquito generation of the year (issued from the first one), hence its potential to be higher than the first one and even partially conceal it. This second peak of higher magnitude would be the cause of the epidemic peak observed on DF incidence during the second half of the year. This epidemic peak would thus be due more to an increase of the number of infected people than to an increase in the mosquito population size and the dengue reproductive ratio. Indeed, dengue epidemic peak appears even when the second peak on the reproductive ratio is not higher than the first one. Such a hypothesis to explain the mechanism of dengue epidemiology in Hanoi can be tested by collecting entomological data (larvae and adult densities estimates) in Hanoi all year-round and translating it into mathematical equations. This would allow to check whether a model based on this hypothesis can generate epidemiological patterns that are in accordance with the ones observed on DF incidence data.

The 2 above-mentioned 6-month cycles observed on time series of DF incidence translate into the sub-annual periodicity that we have characterized in addition to the annual periodicity, with the annual periodicity of DF incidence mostly accounting for the high epidemic peak of the second half of the year, and the sub-annual periodicity mostly accounting for the slow and constant increase of DF incidence of the first half of the year. An interesting result of our analysis is that relative humidity also shows these two annual and sub-annual periodicities and that the sub-annual periodicity of relative humidity leads the sub-annual periodicity of DF incidence by a lag that decreases from 14.30 weeks in 2002 to 5.27 weeks in 2009. As interpreted above, this sub-annual periodicity reflects the first peak of the reproductive ratio that we interpreted in the paragraph above as the first mosquito hatching of the year. Explaining the observed shift in the timing of this first peak by a shift in mosquito hatching is biologically unrealistic. Alternatively, we propose that this shift is due to (i) the building-up of the human population immunity from year to year and (ii) the interactions between dengue serotypes (antibody-dependent enhancement, ADE) as explained below.

Most of primary dengue infections are asymptomatic [1]. Before the emergence of dengue in Hanoi (in 2002), most of the human population may have been susceptible to the 4 dengue serotypes and hence most of the dengue cases may have been primary infections, most likely asymptomatic and thus unnoticed by the surveillance system. As the disease progressively emerges in Hanoi, population immunity to different dengue serotypes increases, thus increasing the number of secondary infections relative to primary ones, and thus increasing the number of symptomatic detected cases. Expected consequences of this mechanism is not only an increase in the number of detected cases from year to year (as visible through the upward trend of DF incidence), but also an earlier detection of the epidemics. The latter would explain this observed shift in the sub-annual periodicity of the DF incidence. This mechanism can potentially be reinforced by some ADE-related mechanisms. Indeed, potential epidemiological consequences of the ADE hypothesis that have been proposed in the literature are that it increases the susceptibility to secondary infections and/or the transmissibility from individuals suffering from secondary infections (see for example [46]). Thus, such a mechanism could also explain the number of detected cases from year to year, the earlier detection of the epidemic, and thus the shift in the sub-annual periodicity of the DF incidence mentioned above. Such a hypothesis could be tested by collecting immunological data from the human population of Hanoi (by an aged-stratified sero-prevalence survey for example) and investigating whether a mathematical model built on this hypothesis does generate the trend in DF incidence mean and timing that we observe on the data.

Both DF incidence and relative humidity exhibit conspicuous annual and sub-annual periodicities and these periodicities happen to be strongly correlated. However, we warn against over-interpretation of such correlations in term of biological causation. One reason for such a caution in particular is that relative humidity is a variable that depends on both absolute humidity and temperature (the former being naturally strongly influenced by rainfalls). In case where absolute humidity (or rainfalls) and temperature are not perfectly correlated (which is most likely the case), we do expect that relative humidity exhibits annual and bi-annual periodicities, as the resultant of two periodic signals that are not perfectly in phase. Thus, instead of looking for a mechanistic link between DF incidence and relative humidity, it may be more relevant to look for two links: (i) one between DF incidence and absolute humidity and (ii) one between DF incidence and temperature, possibly accounting for a possible interaction between the two climatic variables. This particular point will be the topic of a subsequent study.

In conclusion, our analysis on the links between climatic variables and DF incidence in Hanoi raises a number of questions of general interest on the relationships between climate and infectious diseases epidemiology. Because of its highly seasonal climate (and thus potentially highly seasonal dengue transmission too), its important population size and density, and its dengue epidemiological transition (current emergence), Hanoi appears as the ideal set-up to test hypotheses about interaction between serotypes. This is an issue both under-understood and potentially of high relevance for vaccine development. Further investigations on dengue in Hanoi call for additional entomological and immunological data, as well as for theoretical developments.

## References

[1] Bhatt S, Gething PW, Brady OJ, Messina JP, Farlow AW, Moyes CL, Drake JM, Brownstein JS, Hoen AG, Sankoh O, Myers MF, George DB, Jaenisch T, William Wint GR, Simmons CP, Scott TW, Farrar JJ, Hay SI (2013). The global distribution and burden of dengue. Nature.

[2] Tien TKN, Ha DQ, Hien TK, Quang LC (1999). Predictive indicators for forecasting epidemic of dengue/dengue haemorrhagic fever through epidemiological, virological and entomological surveillance. Dengue Bull.

[3] NIHE (2009). Final Report on evaluation of communicable diseases surveillance system in Vietnam 2008.

[4] Ha DQ, Ninh TU (2000). Virological surveillance of dengue haemorrhagic fever in Vietnam, 1987–1999. Dengue Bull.

[5] Cuong HQ, Hien NT, Duong TN, Phong TV, Cam NN, Farrar J, Nam VS, Thai KTD, Horby P (2011). Quantifying the emergence of dengue in Hanoi, Vietnam: 1998–2009. PLoS Negl Trop Dis.

[6] Thai KTD, Cazelles B, Nguyen NV, Vo LT, Boni MF, Farrar J, Simmons CP, Rogier van Doorn H, de Vries PJ (2010). Dengue Dynamics in Binh Thuan Province, Southern Vietnam: Periodicity, Synchronicity and Climate Variability. PLoS Negl Trop Dis.

[7] Vietnamese Ministry of Health (2010). Guidance on declaration, communication, and reporting infectious deseases. Circula.

[8] Toan DTT, Hu W, Thai PQ, Hoat LN, Wright P, Martens P (2013). Hot spot detection and spatio-temporal dispersion of dengue fever in Hanoi, Vietnam. Global Health Action.

[9] Schreiber KV (2001). An investigation of relationships between climate and dengue using a water budgeting technique. Int J Biometeorol.

[10] Hales S, de Wet N, Maindonald J, Woodward A (2002). Potential effect of population and climate changes on global distribution of dengue fever: an empirical model. Lancet.

[11] Nagao Y, Tharava U, Chinumsup P, Tawatsin A, Chansang C, Campbell-Lendrum D (2003). Climatic and social risk factors for Aedes infestation in rural Thailand. Trop Med Int Health.

[12] Depradine C, Lovell E (2004). Climatological variables and the incidence of Dengue fever in Barbados. Int J Environ Health Res.

[13] Promprou S, Jaroensutasinee M, Jaroensutasinee K (2005). Climatic factors affecting dengue haemorrhagic fever incidence in Southern Thailand. Dengue Bulletin.

[14] Promprou S, Jaroensutasinee M, Jaroensutasinee K (2005). Impact of Climatic Factors on Dengue Haemorrhagic Fever Incidence in Southern Thailand. Walailak J Sci & Tech.

[15] Chowell G, Torre CA, Munayco_Escate C, Suarez-Ognio L, Lopez-Cruz R, Hyman JM, Castillo-Chavez C (2008). Spatial and temporal dynamics of dengue fever in Peru: 1994–2006. Epidemiol Infect.

[16] Hsieh YH, Chen CWS (2009). Turning points, reproduction number, and impact of climatological events for multi-wave dengue outbreaks. Trop Med Int Health.

[17] Johansson MA, Dominici F, Glass GE (2009). Local and global effects of climate on dengue transmission in Puerto Rico. PLoS Negl Trop Dis.

[18] Lu L, Lin H, Tian L, Yang W, Sun J, Liu Q (2009). Time series analysis of dengue fever and weather in Guangzhou, China. BMC Public Health.

[19] Hu W, Clements A, William G, Tong S (2010). Dengue fever and El Nino/Southern Oscillation in Queensland, Australia: a time series predictive model. Occup Environ Med.

[20] Wu P-C, Guo H-R, Lung SC, Lin CY, HJa S (2007). Weather as an effective predictor for occurrence of dengue fever in Taiwan. Acta Trop.

[21] Gharbi M, Quenel P, Gustave J, Cassadou S, La Ruche G, Girdary L, Marrama L (2011). Time series analysis of dengue incidence in Guadeloupe, French West Indies: forecasting models using climate variables as predictors. BMC Infect Dis.

[22] Pham HV, Doan HT, Thao TTP, Minh NNT (2011). Ecological factors associated with dengue fever in a Central Highlands province, Vietnam. BMC Infect Dis.

[23] Pinto E, Coelho M, Oliver L, Massad E (2011). The influence of climate variables on dengue in Singapore. Int J Environ Health Res.

[24] Descloux E, Mangeas M, Menkes CE, Lengaigne M, Leroy A, Tehei T, Guillaumot L, Teurlai M, Gourinat AC, Benzler J, Pfannstiel A, Grangeon JP, Degallier N, Lamballerie X (2012). Climate-based models for understanding and forecasting dengue epidemics. PLoS Negl Trop Dis.

[25] Hsieh YH, de Arazoza H, Lounes R (2013). Temporal trends and regional variability of 2001–2002 multiwave DENV-3 epidemic in Havana City: did Hurricane Michelle contribute to its severity?. Trop Med Int Health.

[26] Eastin MD, Delmelle E, Casas I, Wexler J, Self C (2014). Intra- and inter-seasonal autoregressive prediction of dengue outbreaks using local weather and regional climate for a tropical environment in Colombia. Am J Trop Med Hyg.

[27] Yi B, Zhang Z, Xu D, Xi Y, Fu J, Luo J, Yuan M, Liu S, Zuo Z (2003). Relationship of dengue fever epidemic to Aedes density changed by climate factors in Guangdong Province. Wei Sheng Yan Jiu.

[28] Shaman J, Kohn M (2009). Absolute humidity modulates influenza survival, transmission, and seasonality. Proc Natl Acad Sci U S A.

[29] Vietnamese Ministry of Health (2006). Guidelines for Surveillance, Prevention and Control of Dengue and Dengue Haemorrhagic Fever. Circula.

[30] Wallace JM, Hobbs PV (2006). Atmospheric Science, An Introductory Survey. Academic New York.

[31] Cazelles B, Chavez M, McMichael AJ, Hales S (2005). Nonstationary influence of El Niño on the synchronous dengue epidemics in Thailand. PLoS Med.

[32] Cazelles B, Hales S (2006). Infectious Diseases, Climate Influences, and Nonstationarity. PLoS Med.

[33] Simões TC, Codeço CT, Nobre AA, Eiras AE (2013). Modeling the Non-Stationary Climate Dependent Temporal Dynamics of Aedes aegypti. PLoS One.

[34] Cazelles B, Chavez M, Constantin de Magny G, Guegan JF, Hales S (2007). Time-dependent spectral analysis of epidemiological time-series with wavelets. J R Soc Interface.

[35] Torrence C, Compo GP (1998). A practical guide to wavelet analysis. Bull Am Meteorol Soc.

[36] Soper HE (1929). The Interpretation of Periodicity in Disease Prevalence. Roy Stat Soc A.

[37] Keeling MJ, Rohani P (2008). Modeling Infectious Diseases in Humans and Animals. Princeton University Press.

[38] Andraud M, Hens N, Marais C, Beutels P (2012). Dynamic epidemiological models for dengue transmission: a systematic review of structural approaches. PLoS One.

[39] RCoreTeam (2012). R: A Language and Environment for Statistical Computing R.

[40] Gouhier T (2013). Biwavelet: Conduct univariate and bivariate wavelet analyses.

[41] Center for Excellence in Disaster Management and Humanitarian Assisstance (2008). Dengue fever cases rise after floods plague Vietnam.

[42] Thu HM, Aye KM, Thein S (1998). The effect of temperature and humidity on dengue virus propagation in Aedes aegypti mosquitoes. Southeast Asian J Trop Med Public Health.

[43] Tipayamongkholgul M, Fang C-T, Klinchan S, Liu CM, King CC (2009). Effects of the El Niño-Southern Oscillation on dengue epidemics in Thailand, 1996–2005. BMC Public Health.

[44] Wongkoon S, Jaroensutasinee M, Jaroensutasinee K (2011). Climatic variability and dengue virus transmission in Chiang Rai, Thailand. Biomedica.

[45] Higa Y, Yen NT, Kawada H, Son TH, Hoa NT, Takagi M (2010). Geographic Distribution of Aedes aegypti and Aedes albopicuts Collected from Used Tires in Vietnam. Am Mosq Control Assoc.

[46] Recker M, Blyuss KB, Simmons CP, Hien TT, Wills B, Farrar J, Gupta S (2009). Immunological serotype interactions and their effect on the epidemiological pattern of dengue. Proc R Soc B.

[CR47] The pre-publication history for this paper can be accessed here: http://www.biomedcentral.com/1471-2458/14/1078/prepub

